# Antigen-specific antibody Fc glycosylation enhances humoral immunity via the recruitment of complement

**DOI:** 10.1126/sciimmunol.aat7796

**Published:** 2018-08-17

**Authors:** Giuseppe Lofano, Matthew J. Gorman, Ashraf S. Yousif, Wen-Han Yu, Julie M. Fox, Anne-Sophie Dugast, Margaret E. Ackerman, Todd J. Suscovich, Joshua Weiner, Dan Barouch, Hendrik Streeck, Susan Little, Davey Smith, Douglas Richman, Douglas Lauffenburger, Bruce D. Walker, Michael S. Diamond, Galit Alter

**Affiliations:** 1 Ragon Institute of MGH, MIT and Harvard, Cambridge, MA 02139, USA; 2 Department of Immunology and Biotechnology, Tropical Medicine Research Institute, Khartoum, Sudan; 3 Department of Biological Engineering, Massachusetts Institute of Technology, Cambridge, MA 02142, USA; 4 Departments of Medicine, Molecular Microbiology, and Pathology & Immunology, Washington University School of Medicine, St. Louis, MO 63110, USA; 5 Thayer School of Engineering, Dartmouth College, Hanover, NH 03755, USA; 6 Center for Virology and Vaccine Research, Beth Israel Deaconess Medical Center, Boston, MA 02115, USA; 7 Institut für HIV Forschung, Universität Duisburg-Essen, Essen, Germany; 8 University of California, San Diego, San Diego, CA 92093, USA; 9 VA San Diego Healthcare System, San Diego, CA 92161, USA; 10 Institute for Medical Engineering and Science, Massachusetts Institute of Technology, Cambridge, MA 02139, USA; 11 Howard Hughes Medical Institute, Chevy Chase, MD 20815, USA

## Abstract

HIV-specific broadly neutralizing antibodies (bNAbs) confer protection after passive immunization, but the immunological mechanisms that drive their development are poorly understood. Structural features of bNAbs indicate that they originate from extensive germinal center (GC) selection, which relies on persistent GC activity. However, why a fraction of infected individuals are able to successfully drive more effective affinity maturation is unclear. Delivery of antigens in the form of antibody-immune complexes (ICs), which bind to complement receptors (CRs) or Fc receptors (FcRs) on follicular dendritic cells, represents an effective mechanism for antigen delivery to the GC. We sought to define whether IC-FcR or CR interactions differ among individuals who develop bNAb responses to HIV. Enhanced Fc effector functions and FcR/CR interactions, via altered Fc glycosylation profiles, were observed among individuals with neutralizing antibody responses to HIV compared with those without neutralizing antibody activity. Moreover, both polyclonal neutralizer ICs and monoclonal IC mimics of neutralizer antibodies induced higher antibody titers, higher-avidity antibodies, and expanded GC B cell reactions after immunization of mice via accelerated antigen deposition within B cell follicles in a complement-dependent manner. Thus, these data point to a direct role for altered Fc profile/complement interactions in shaping the maturation of the humoral immune response, providing insights into how GC activity may be enhanced to drive affinity maturation in next-generation vaccine approaches.

## INTRODUCTION

The development of a protective vaccine against HIV will likely require the induction of highly cross-reactive broadly neutralizing antibodies (bNAbs). Although current vaccination regimens can readily induce Abs capable of neutralizing autologous viruses (*1*, *2*), these immunization strategies have generated only weakly cross-neutralizing (heterologous) Abs (*3*, *4*). Although vaccination has failed to induce Abs with appreciable neutralization breadth, 5 to 30% of infected individuals naturally develop bNAbs, albeit after several years of infection (*5*, *6*) and one step behind the autologous virus (*7*). Thus, elucidating the immunological processes that allow some individuals to overcome the immunologic barrier to developing neutralizing breadth may provide critical insights for the development of a vaccine capable of inducing bNAbs.

Although our understanding of the molecular and structural features of Abs that allow for broad neutralization has increased markedly over the past decade (*8*, *9*), the underlying immunological pathways that lead to these unusual features have yet to be fully elucidated. Nearly all bNAbs develop after extensive somatic hypermutation and often contain mutated framework regions (*9*, *10*), features that are indicative of the prolonged affinity maturation. Previous studies have linked high viral loads, low CD4 T cell counts, and immune activation in the evolution of bNAbs (*11*–*14*). In the setting of low levels of viremia and high CD4 counts, bNAb activity still evolves, but only in the setting of a unique plasma type I interferon and germinal center (GC)–centric cytokine signature, pointing to the need for persistent GC activity (*15*). Yet, how these individuals maintain GC activity, particularly in the setting of low antigenic loads, is unclear.

Antigen delivery to the lymph node, and specifically to follicular dendritic cells (FDCs), plays a key role in both initiating and sustaining the GC reaction (*16*). Specifically, antigen may be delivered to the lymph node via both “naked” antigen diffusion from the lymph or active antigen delivery via the delivery of antigen in the form of immune complexes (ICs) by noncognate B cells or local macrophages (*17*–*20*). Although HIV antigens likely arrive in the GC in the absence of Ab opsonization in acute HIV infection, high titers of HIV-specific Abs are induced over the course of infection that contribute to IC formation (*21*, *22*), antigen capture and delivery, and seeding of antigens in the GC (*23*). However, whether differences in IC quality could contribute to GC generation and persistence, affinity maturation, and the evolution of neutralization breadth is unknown.

IC activity is modulated by both the Fab and Fc domains of the Ab. The Fab drives antigen specificity, whereas the Fc region determines which innate immune receptors, including Fc receptors (FcRs) and complement receptors (CRs), ICs can bind. Depending on the FcRs and CRs engaged by an IC, antigens can be rapidly destroyed or transported to the GC for antigen presentation (*18*, *19*, *24*–*27*). Hence, modifications to the Fc domain, including changes in Ab isotype/subclass and glycosylation (*28*–*31*), may have a profound impact on IC delivery via improved IC affinity for particular FcRs/CRs. Along these lines, influenza-specific Fc Ab glycosylation after vaccination is associated with enhanced affinity maturation of the influenza-specific response (*32*), highlighting the potential influence of the Fc domain changes after vaccination on the antiviral activity of the vaccine-induced immune response.

We speculated that qualitative changes in the Fc domain track with the development of bNAbs during HIV infection and point to the mechanism by which the Fc domain of the Ab may be leveraged to drive the evolution of highly affinity-matured bNab responses. Using “systems serology” (*33*), we observed significant differences in the Fc profiles of HIV-specific Abs in individuals who developed neutralizing breadth (neutralizers) compared with those who did not (non-neutralizers). Moreover, immunization with ICs generated with polyclonal Abs from neutralizers induced higher HIV-specific Ab responses after vaccination. This biological activity was linked to particular Ab glycosylation profiles, which, when replicated with monoclonal IC immunization, resulted in enhanced IC capture by noncognate B cells, accelerated IC deposition within B cell follicles, and elevated Ab titers and avidity Ab production in a complement-dependent manner. Collectively, these data point to Fc glycosylation as a direct modulator of B cell immunity and suggest an approach by which next-generation vaccines can exploit both ends of the Ab to drive enhanced B cell affinity maturation that may be required against highly mutable targets.

## RESULTS

### Selective Fc functional profile differences exist among neutralizers

Given previous associations of the evolution of neutralizing Ab breadth in the setting of high immune activation, low CD4 counts, and high viral loads (*12*, *14*), we aimed to dissect the potential involvement of changes in IC biology in a group of spontaneous controllers of HIV (≤2000 copies/ml), a fraction of whom developed bNAb responses, despite the absence of high levels of plasma antigenemia (*15*). Despite lower viremia, previous studies demonstrated that equal numbers of controllers evolve neutralization breadth (*15*) compared with normal progressors (*12*, *14*). Seventy-one participants were included in this study, with neutralization breadth ranging from 9 to 100% coverage of a panel of 11 tier 2/3 viruses (neutralizers) that were compared with a matched group of 60 participants with no evidence of neutralizing Ab breadth (0% neutralizing activity across 11 tier 2 and tier 3 viruses; non-neutralizers). The two groups were matched for duration of infection, viral loads, and CD4 counts.

**Fig. 1:**
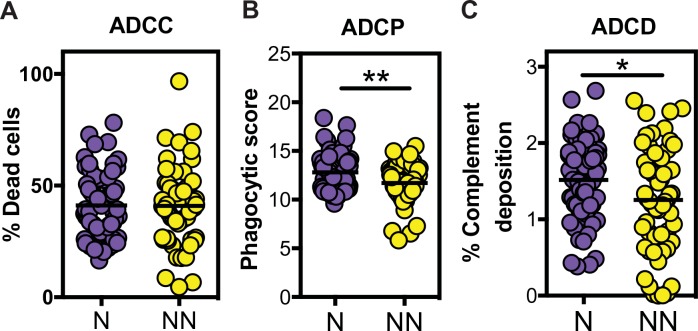
**Unique Fc functional profiling of HIV-specific Abs from neutralizers.** (**A** to **C**) Serum Abs from neutralizers (N; purple) and non-neutralizers (NN; yellow) were evaluated for their ability to promote NK-dependent ADCC against gp120-pulsed CD4^+^ T cells (A), monocyte-directed phagocytosis of gp120-functionalized fluorescent beads (B), or complement deposition (C3b) on the surface of gp120-pulsed CD4^+^ target cells (C). All assays were performed in duplicate or triplicate across all 131 individuals. An unpaired *t* test was used for statistical analysis. *P < 0.05 and **P < 0.01. The horizontal bars in all panels indicate mean.

No differences in the ability of Abs to recruit natural killer (NK) cells to kill HIV envelope–coated target cells in an Ab-dependent cellular cytotoxicity (ADCC) assay were observed between the neutralizers and non-neutralizers (Fig. 1A). In contrast, significant differences were observed in the capacity of neutralizer Abs to direct Ab-dependent cellular phagocytosis (ADCP) and Ab-dependent complement deposition (ADCD) (Fig. 1, B and C). Given the use of different FcRs in driving distinct innate functions, these data suggested that neutralizer Abs were selectively skewed to drive enhanced phagocytic and complement-depositing activity, but not NK cell cytotoxicity.

**Fig. 2:**
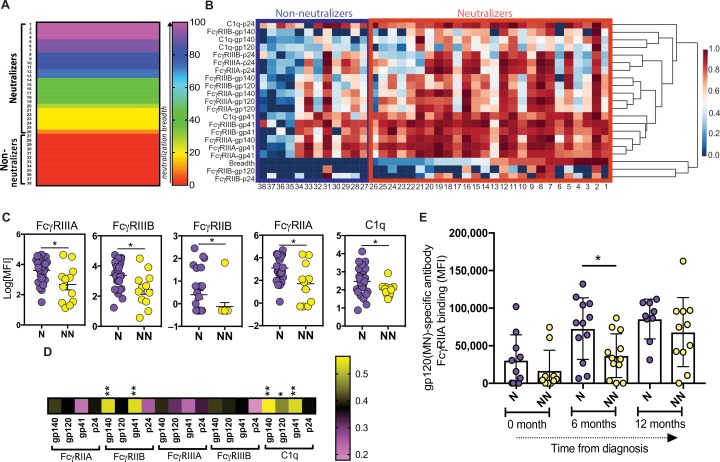
**Enhanced, but selective, FcR and complement binding by HIV-specific Abs in neutralizers.** (**A**) The rainbow heat map shows the breadth of neutralization and sample ID number across the 26 neutralizers and 12 non-neutralizers that were profiled more deeply in this and subsequent figures. (**B**) The larger heat map illustrates the binding capacity (MFI) of gp140-, gp120-, gp41-, and p24-specific serum Abs to FcγRIIA, FcγRIIB, FcγRIIIA, FcγRIIIB, and C1q protein. Row 19 represents the breadth of neutralization. Each additional row represents the binding level for a single antigen specificity to a single FcR/C1q. Each column represents one individual (sample ID number is below the heat map), either a neutralizer (red box) or a non-neutralizer (blue box). Data were normalized across rows. The scale bar represents the *Z*-normalized scores. (**C**) The dot plot represents the representative univariate binding of gp120-specific Abs from each group to FcγRIIA, FcγRIIB, FcγRIIIA, FcγRIIIB, and C1q. (**D**) The heat map strip highlights the correlation between gp140-, gp120-, gp41-, and p24-specific Ab binding to each FcR/C1q and the breadth of neutralization across all neutralizers and non-neutralizers. (**E**) The hybrid dot/bar plot shows the evolution of gp120-specific FcγRIIA binding Abs in the first year after infection in a group of acutely infected HIV participants, half of which went on to develop bNAbs (purple) or not (yellow). Each dot represents one individual, with neutralizers (N) in violet and non-neutralizers (NN) in yellow. A Mann-Whitney test was used for statistical analysis to compare groups in (C). An ANOVA, with a post hoc Tukey’s test, was used to compare between groups across time points in (E). A Spearman correlation with a post hoc Bonferroni correction was used to examine the relationship between Fc profiles and breadth of neutralization (D). *P < 0.05 and **P < 0.01. The horizontal bars in all panels indicate mean. Error bars represent standard error of mean (SEM) in (C) and standard deviation (SD) in (E).

To further dissect the specific FcRs that were selectively recruited by neutralizer Abs, we used a multiparametric Luminex assay to measure differences in IC binding to a spectrum of Fcγ receptors (FcγRs) and complement proteins. With a panel of HIV envelope–conjugated Luminex beads, ICs were formed, and binding to the ICs by FcγRs and complement was measured. A subset of the original cohort, composed of 38 individuals broadly covering a range of neutralization breadths (0 to 100%) (Fig. 2A and table S1), for whom sufficient plasma was available, was included in this analysis. The individuals included 26 participants who neutralized between 9 and 100% of the 11 tested tier 2/3 viruses and 12 participants with no evidence of neutralizing Ab breadth (Fig. 2A and table S1). In addition, viral loads, CD4 counts, days after diagnosis, and distribution of ADCD, ADCC, and ADCP responses were similar between the groups. Marked differences were observed among the neutralizer and non-neutralizer Abs in their ability to bind to C1q and FcγRs (Fig. 2B). Specifically, a higher proportion of neutralizers exhibited enhanced gp41-specific FcR binding and enhanced gp120 and gp140 binding to FcγRIIIA and FcγRIIIB, and a select set of neutralizers also showed stronger binding to C1q (Fig. 2B). Univariate analysis highlighted the overall elevated levels of FcγR/C1q binding Abs among neutralizers (Fig. 2C). Moreover, positive associations between breadth and FcR/C1q binding were observed across all HIV-specific Ab responses; however, only gp140- and gp41-specific Ab binding to FcγRIIB and C1q was statistically significant (Fig. 2D). These data suggest an overall enhanced FcγRs and C1q binding profile in Abs from neutralizers compared with non-neutralizers, consistent with enhanced ADCD and ADCP (Fig. 1). Furthermore, previous longitudinal analysis pointed to significantly enhanced Fc function and receptor binding among individuals who went on to generate neutralizing responses before the evolution of the neutralizing Abs (*34*). Likewise, analysis of plasma samples from a separate cohort of acutely infected individuals, half of whom went on to generate neutralizing Abs, pointed to selectively elevated gp120-specific FcγRIIA binding preceding the evolution of bNAbs after 2 years of infection (Fig. 2E) (*7*). Given the importance of both FcγR and C1q in the delivery of IC cargo to the GC (*17*, *35*, *36*), these data point to unique IC profiles among neutralizers with a higher intrinsic capacity to interact with Fc-binding proteins involved in antigen delivery to the GC, thereby potentially contributing to persistent GC activity and enhanced affinity maturation.

### HIV-specific Abs from neutralizers promote GC reactions

**Fig. 3:**
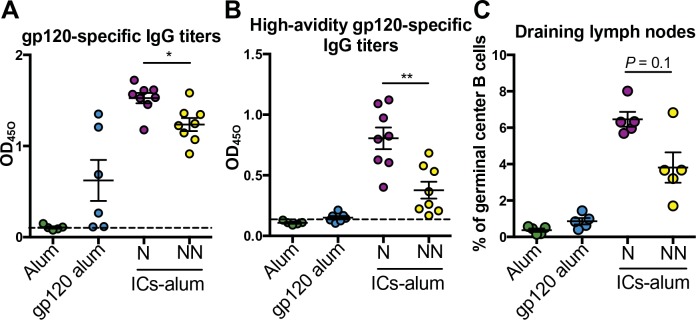
**Neutralizer ICs drive higher-avidity Abs and expanded GC B cell responses.** (**A** to **C**) BALB/c mice were immunized twice, 3 weeks apart, with alum-adjuvanted gp120 ICs (ICs-alum) generated with serum Abs from four neutralizers (N) or four non-neutralizers (NN) (*n* = 5 to 8 mice per group, two experiments). Control groups received alum-adjuvanted gp120 or adjuvant alone. Ten days after the last immunization, we measured the titers of gp120-specific IgG Abs (A), the titers of high-avidity gp120-specific IgG Abs (B) in sera, and the percentage of GC B cells (C), defined as live CD3^−^CD19^+^CD38^−^CD95^+^ cells, in draining lymph nodes of immune mice. All assays were run in duplicate, and a Mann-Whitney test was used to compare responses induced by neutralizer (N) or non-neutralizer (NN) immunized animals. *P < 0.05 and **P < 0.01. The horizontal bars in all panels indicate mean, and error bars in all panels represent SEM.

To test the hypothesis that differences in Fc profiles generated in neutralizers and non-neutralizers were responsible for differences in GC reactions and affinity maturation, we generated ICs from neutralizers and non-neutralizers. Ab pools were generated from four neutralizers and four non-neutralizers, matched for gp120-specific Ab titers and avidity (fig. S1A). Specifically, recombinant HIV gp120 proteins were complexed with polyclonal Abs from neutralizer and non-neutralizer pools at Ab concentrations aimed at achieving equivalent IC occupancy (fig. S1, B and C). All complexes were co-delivered to mice with alum as a baseline comparator to standard immunization. Thus, equivalent amounts of alum-adjuvanted ICs, alum-adjuvanted gp120, or alum alone were administered. Mice received two immunizations, 3 weeks apart, and 10 days after the last immunization, gp120-specific Ab serum titers, high-avidity gp120-specific Ab titers, and the frequency of GC B cells in the draining lymph nodes of immunized mice were measured. As expected, higher levels of gp120-specific Abs were induced in IC-immunized mice compared with mice vaccinated with antigen alone (Fig. 3A). Mice immunized with the ICs from neutralizers demonstrated slightly higher levels of overall Ab titers and more avid Abs compared with the mice vaccinated with ICs from non-neutralizers (*P* < 0.05; Fig. 3, A and B). Furthermore, higher frequencies of GC (CD19^+^CD30^−^CD95^+^) B cells were observed after immunization with ICs from neutralizers compared with mice immunized with ICs from non-neutralizers (Fig. 3C). Given the overlap in mouse and human FcR responses to human monoclonal Abs (mAbs) (*37*), suggesting sufficient cross-species interaction, these data suggest that the Fc differences in neutralizer Abs may not only represent a biomarker of a more effective humoral immune response but also contribute to differences in GC activity.

### Unique antigen-specific Ab glycosylation and elevated immunoglobulin G1 are enriched among neutralizers

**Fig. 4:**
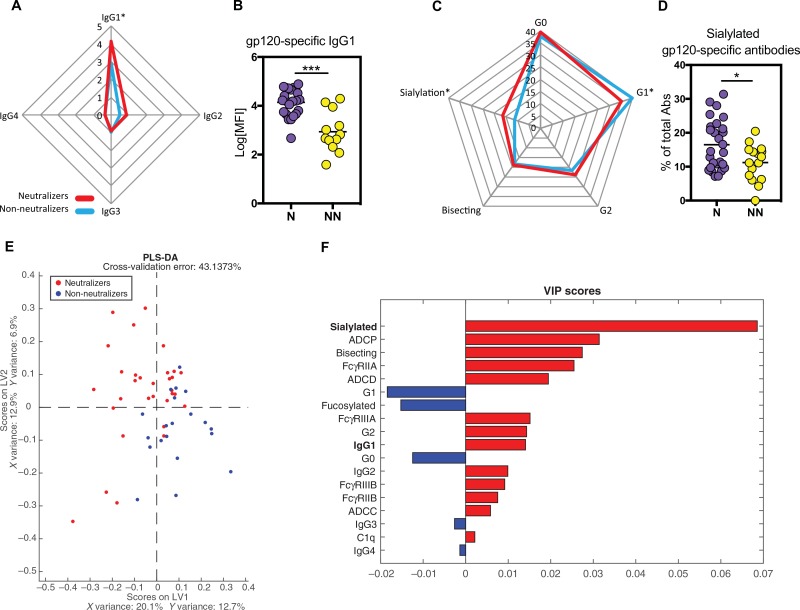
**Sialylated IgG1 gp120-specific Abs are increased in neutralizers.** (**A**) The radar plot shows the relative differences in gp120-specific IgG1, IgG2, IgG3, and IgG4 Ab titers in sera from neutralizers (N; red; *n* = 26) or non-neutralizers (NN; blue; *n* = 12). (**B**) The dot plot shows the gp120-specific IgG1 Ab titer differences across the neutralizers and non-neutralizers. (**C**) The radar plot describes the relative distribution of gp120-specific IgG Ab glycan levels in sera from neutralizers (N; red) or non-neutralizers (NN; blue). (**D**) The dot plot illustrates the percentage of sialylated gp120-specific IgG Abs among the groups. (**E**) The PLS-DA, using all Fc profile data including gp120-specific Ab titers, glycan profiles, binding to FcRs and complement proteins, ADCP, ADCD, and ADCC, was used to separate the two groups. Each dot represents a neutralizer (red) or non-neutralizer (blue). (**F**) The bar graph shows the variable importance in projection (VIP) score rank for the minimal Ab features that were used in the PLS-DA to separate the groups. As few as 17 of the total features collected across the cohort were required to nearly completely separate out the neutralizers and non-neutralizers. The variables were ranked in their importance in driving separation in the model, with the largest bars representing the most important contributions and the smallest bars representing more minimal contributions. The direction and color of the bars indicate whether the feature was enriched in the neutralizers (red) or in the non-neutralizers (blue). A Mann-Whitney test was used for statistical analysis in (B) and (D). *P < 0.05 and ***P < 0.001. The horizontal bars in all panels indicate mean, and error bars in all panels represent SEM.

To define the biophysical differences in neutralizer and non-neutralizer IC profiles that may have contributed to enhanced humoral immune responses, we interrogated differences in both immunoglobulin G (IgG) subclass selection levels and Fc glycosylation profiles on HIV-specific Abs by capillary electrophoresis. Although no differences were observed in antigen-specific IgG2, IgG3, and IgG4 levels across the two groups, neutralizers had higher HIV-specific IgG1 titers to all HIV antigens, but not to influenza hemagglutinin (HA) (Fig. 4, A and B, and fig. S2A), highlighting the infection-specific change in Ab levels. Elevated HIV-specific IgG responses were not linked directly to viral load, days after diagnosis, or viremic or elite controller status, but did negatively correlate with CD4 count (fig. S3, A to D) (*14*, *37*, *38*). However, as previously reported, a trend was observed in neutralization breadth and CD4 counts and viral load (fig. S3, E and F) (*12*, *14*, *38*). Thus, differences in IC activity may be attributable to higher overall HIV-specific IgG1 levels, which could account for improved overall FcR binding. However, selectively enhanced binding to C1q and FcγRII (Fig. 2C) and immunization differences using matched Ab-bound complexes (Fig. 3) could not be explained by elevated IgG1 levels or differential subclass selection profiles.

Given the emerging appreciation for the role of antigen-specific Ab glycoforms in shaping Fc activity (*28*, *29*), we profiled HIV-specific Ab glycosylation (*39*). Using standard capillary electrophoresis (*39*) on equivalent amounts of enzymatically collected Fc glycans from neutralizers and non-neutralizers, we observed elevated levels of sialylated glycan structures on HIV envelope–specific IgG-Fcs from neutralizers compared with non-neutralizers. In contrast, non-neutralizers had higher levels of singly galactosylated (G1) structures on their HIV-specific IgG1 Abs (Fig. 4, C and D). Thus, selective differences in sialylation and galactosylation were observed among neutralizers and non-neutralizers, pointing to qualitative differences in IgG1 Ab after translational modifications.

To further define the minimal HIV-specific features that were selectively enriched among neutralizers, we used an orthogonal multivariate partial least square discriminant analysis PLS-DA combining all gp120-specific features. Using all Fc profile features, we observed nearly complete separation between neutralizers and non-neutralizers (Fig. 4E). The dominant features that selectively split the two groups included the level of sialylated glycan structures and overall levels of gp120-specific IgG1 Abs, with the percentage of sialylated gp120-specific Abs ranking as the top distinguishing feature (Fig. 4, E and F). Consistent with previous studies pointing to an association between the levels of vaccine-induced sialylated Abs and Ab affinity maturation (*32*), sialylated HIV-specific Abs were selectively enriched among neutralizers, pointing to a potential link between posttranslational Ab sialylation and affinity maturation across disease and vaccination. However, whether sialylation represents a biomarker of a more highly affinity-matured immune response or directly contributes to affinity maturation remains unknown.

### HIV Ab glycoforms modulate IC-driven innate and adaptive immunity

**Fig. 5:**
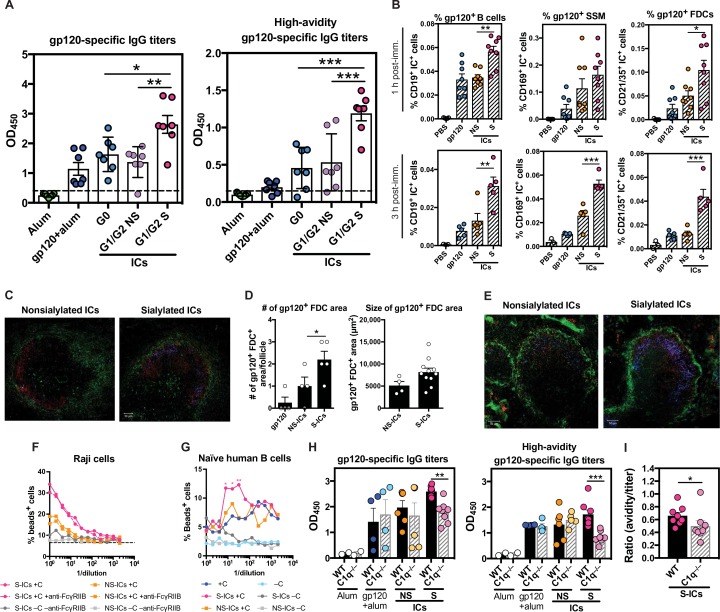
**Sialylated Ab-ICs promote enhanced humoral immune responses.** (**A**) The dot plots show gp120-specific Ab titers (left) or avid Ab titers (right) 10 days after the second immunization in BALB/c mice receiving alum alone (green), alum-adjuvanted gp120 (blue), agalactosylated PGT121 (G0-ICs) with alum (aqua), nonsialylated galactosylated PGT121 (G1/G2 NS-ICs) with alum (lavender), or sialylated galactosylated PGT121 (G1/G2 S-ICs) with alum (pink) (*n* = 3 to 8 mice, two experiments). (**B**) The dot plots highlight the number of fluorescently labeled ICs on noncognate B cells (gp120^+^ B cells), macrophages (gp120^+^ SSM), or FDCs (gp120^+^ FDCs) from draining lymph node at 1 and 3 hours after injection with nonsialylated IC (NS-IC) or sialylated IC (S-IC) (*n* = 4 to 8 mice, two experiments). (**C**) The confocal microscopy images depict the level of IC deposition in a B cell follicle 1 hour after a mouse is immunized with NS-ICs (left) or S-ICs (right) and stained for g120 (blue), macrophages (green), and GL-7 (red). Representative image of three images from two to three mice per condition. (**D**) The bar graphs show the number (left) and area occupied (right) of gp120^+^bead^+^ FDCs in a draining lymph node. (**E**) The confocal microscopy images depict IC deposition in a B cell follicle 3 hours after immunization and stained with the same targets as (C). Representative image of three images from two to three mice per condition. (**F** and **G**) The line graphs demonstrate the percentage of bound Raji or naïve human B cells after incubation with APC bead–labeled NS-ICs (yellow) or S-ICs (pink), with (colored line) or without complement (gray line), with (blue) or without complement (light blue) alone, or α-FcγRIIB blocking Abs (hatched lines) (F, *n* = 2; G, *n* =3). (**H**) The dot plots show the gp120-specific IgG Ab titers (left) and high-avidity gp120-specific Ab titers (right) after immunizing WT or *C1q* ^−/−^ C57BL/6 mice (*n* = 2 to 8, two experiments) with alum (white), gp120-alum (blue), NS-ICs with alum (yellow), or S-ICs with alum (pink). (**I**) The dot plot highlights the ratio of high-avidity gp120-specific Abs to total gp120-specific Abs in WT (dark pink) versus *C1q* ^−/−^ (light pink) mice after immunization with S-ICs. All ELISAs were performed in triplicate, and a one-way ANOVA followed by Tukey’s post test was used for (A), (B), and (D). Mann-Whitney and one-way ANOVA with Holm-Sidak’s multiple comparisons test were used for (H). Two-way ANOVA with Dunnett’s correction comparing samples with the negative control (no Ab, no complement) was used for (G). Mann-Whitney test was used in (I). *P < 0.05, **P < 0.01, and ***P < 0.001. The horizontal bars in all panels indicate mean. Error bars represent SEM in (B), (D), (H), and (I) and SD in (A).

To define the involvement of sialylated Abs in driving affinity maturation and to separate the role of sialylation from Ab titer differences between neutralizers and non-neutralizers (fig. S1A), we immunized animals with ICs generated with a single mAb, but with differential glycosylation. ICs were generated with an HIV-specific mAb (PGT121) that was fractionated and enzymatically prepared into three separate Ab Fc fractions to generate agalactosylated ICs (G0-ICs), nonsialylated galactosylated ICs (G1/G2 NS-ICs), or sialylated ICs (G1/G2 S-ICs). Despite the presence of a glycan in the PGT121 Fab (*40*), removal of sialylation did not affect PGT121 binding or affinity (fig. S4, A to D). Mice were immunized with alum-formulated ICs, alum-formulated antigen alone, or alum alone, and Ab levels, avidity, and B cell frequencies were interrogated 10 days after the second immunization. Mice immunized with S-ICs exhibited increased gp120-specific IgG titers compared with those immunized with NS-ICs, G0-ICs, or gp120 alone (Fig. 5A). Moreover, immunization with S-ICs also induced more avid gp120-specific Abs compared with all other immunized groups (Fig. 5A). Although the Fab domain may also be sialylated (*41*), such as in PGT121 (*40*), only the Fc glycan contributes to FcR/complement interactions in an indirect manner by reshaping the Ab Fc to tune FcR/complement engagement (*42*). These data implicate Fc sialylation as a direct modulator of the humoral immune response, rather than a simple biomarker of an affinity-matured response.

### Sialylated ICs drive enhanced antigen deposition

To begin to dissect the mechanism by which S-ICs contribute to enhanced B cell affinity maturation, we examined the distribution of these ICs early after immunization. A number of cells have been implicated in IC capture and transfer to GCs, including sinusoidal macrophages (SSMs) and noncognate B cells that collectively transfer ICs to FDCs. Glycan-fractionated PGT121-ICs were prepared with a fluorescently labeled gp120. The ICs were delivered intravenously, and IC capture was measured by flow cytometry and immunofluorescent microscopy 1 and 3 hours after IC administration. After 1 hour, significantly higher frequencies of ICs were observed on B cells and FDCs in the presence of S-ICs compared with NS-ICs or antigen alone (Fig. 5B); after 3 hours, SSMs, B cells, and FDCs captured higher levels of ICs in the presence of sialylated Abs.

To visualize the localization of the ICs after delivery, we performed fluorescence microscopy on splenic follicles. One hour after vaccination, follicles from mice receiving the sialylated ICs exhibited greater IC deposition within the follicle than those receiving the nonsialylated ICs (Fig. 5C). Moreover, S-IC deposition was proximal to FDCs, whereas NS-IC deposition, though infrequent, was observed closer to SSMs. Furthermore, vaccination with the sialylated ICs induced higher numbers of gp120^+^ FDC areas per follicle, two on average, and of larger size of the areas, compared with vaccination with nonsialylated ICs (Fig. 5D). Furthermore, after 3 hours, greater antigen deposition was observed in the presence of S-ICs compared with NS-ICs, colocalizing with GL-7^+^ GC B cells (Fig. 5E). These data suggest that S-ICs may contribute to enhanced humoral immune activity via the rapid and effective deposition of ICs within GCs via enhanced IC capture and delivery to the follicle. How sialylated ICs are selectively captured and delivered to the follicle remains unknown.

### Sialylated Fc antigen effects are complement-dependent

Emerging data suggest that complement has an essential role in IC-mediated antigen deposition on FDCs (*17*). We assessed whether uptake of sialylated ICs depended on complement. Using a noncognate reporter B cell line, Raji cells, expressing both complement and FcRs (*43*–*45*), we assessed the capture of S-ICs and NS-ICs in the presence or absence of complement and/or FcγRIIB blockade by flow cytometry. As expected, at the highest concentration, both S- and NS-ICs were trapped by the noncognate B cells in the presence of complement; however, S-ICs bound at higher levels in the presence of complement compared with NS-ICs. In contrast, no difference was observed between S- and NS-ICs in the setting of FcγRIIB blockade (Fig. 5F). Increased binding of S-ICs compared with NS-ICs, in the presence of complement, was confirmed on naïve primary human B cells (Fig. 5G). These data suggest a dominant role for S-IC interaction with complement in IC capture.

To confirm the interaction between S-ICs and complement, we performed IC-based immunizations in complement-deficient mice. Wild-type (WT) or *C1q*-deficient (*C1q* ^−/−^) congenic mice were immunized twice with the ICs or antigen alone. As expected, antigen-specific Ab responses were highest in the WT mice immunized with S-ICs, with intermediate levels observed in WT mice vaccinated with NS-ICs or antigen alone (Fig. 5H). However, a significant reduction in Ab titers and avidity were observed in the *C1q*^−/−^ mice vaccinated with S-ICs compared with WT mice (Fig. 5H). Moreover, whereas all animals appeared to reach a plateau after the second boost, the *C1q*^−/−^ mice immunized with S-ICs exhibited lower Ab titers and avidity compared with WT counterparts (Fig. 5, H and I). These data provide further evidence to support a role in Ab sialylation in complement-dependent IC delivery for B cell priming and boosting. Although these data do not preclude a role for other FcRs in IC-mediated antigen deposition in the lymph node, they support a critical role for sialylated Ab-induced complement deposition as a driver of high-affinity B cell responses.

## DISCUSSION

Although efforts in vaccine design to induce bNAbs continue to struggle to drive broad tier 2 heterologous immunity, our evolving appreciation for the unique immunological features of the broad cross-tier neutralizing breadth that emerges naturally in a select population of infected individuals provides unexpected insights for the rational development of a protective vaccine. Here, we observed that individuals who developed bNAbs had HIV-specific Abs with enhanced CR and FcγR binding, selectively skewed to induce phagocytosis and complement deposition via enhanced HIV-specific Ab Fc domain sialylation. Given the importance of Fc binding for antigen deposition in the GC and our emerging appreciation for enhanced and persistent GC activation among individuals with neutralizing Abs (*10*, *12*, *15*, *46*), we speculated that the enhanced Fc functionality could actively contribute to the evolution of HIV-specific neutralization breadth. ICs generated with either Abs from individuals with these enhanced Fc functions or highly sialylated mAbs induced higher titers of more avidly binding Abs after IC immunization. Thus, these data suggest that the Fc domain of the Ab may modulate the formation of the GC reaction, catalyzing the persistent selection and affinity maturation of B cells required to drive the unusual B cell selection associated with the development of bNAbs against HIV.

Rational vaccine design has led to the creation of both stabilized trimers and minimal scaffolds able to focus the immune response to sites of neutralization vulnerability (*8*, *47*, *48*). However, vaccination with these constructs continue to induce Abs with narrow breadth, likely due to a limited amount of affinity maturation. This has led the field to explore novel immunization strategies and alternate adjuvants, as well as to focus vaccine design on eliciting bNAbs in the absence of high levels of somatic hypermutation (*6*, *49*, *50*). Our results suggest that naturally modified Abs can selectively augment affinity maturation via the rapid capture and delivery of antigens to the GC. These data are consistent with studies of influenza vaccination, where the level of HA-specific Ab sialylation was associated with the extent of affinity maturation (*32*). In our study, the administration of selectively glycosylated Abs at the time of antigen exposure led to a marked increase in antigen deposition and maturation of high-avidity Abs, pointing to a role for antigen-specific Ab glycosylation as a regulator of GC activity. Whereas Fab glycosylation is more uniform (*41*) and not involved in Fc/FcR interactions (*42*), mounting data suggest that Ab Fc glycosylation is programmed in an antigen-specific manner (*51*), modulated by vaccination (*51*, *52*), and tuned by adjuvants (*53*), which offers an opportunity for manipulating the quality of innate immune–recruiting Abs to enhance immunity upon boosting.

Beyond the role of immune activation, low CD4 T cell counts, and high viral loads as drivers of neutralizing Ab breadth, recent data suggest that individuals who generate bNAbs have autoimmune-like transcriptional signatures (*54*–*56*). These transcriptional programs may enable B cells to break the necessary tolerance to evolve polyreactive B cell receptors and drive extensive affinity maturation (*5*, *56*, *57*), even within framework regions that are rarely targeted for somatic mutation in healthy B cells (*10*). Similarly, IC levels and signaling have been linked to autoimmune signatures in B cells in individuals with rheumatoid arthritis and lupus (*58*–*60*). However, whether neutralizers have autoimmune-like signatures due to intrinsic differences in their B cell populations or due to the higher abundance of ICs that can interact with FcRs and CRs is unknown. Nevertheless, it is plausible that increased IC signaling could lead not only to enhanced antigen deposition but also to increased B cell activation, enabling antigen-specific B cells to undergo more extensive maturation, tolerate more mutations, and even develop responses to cross-reactive epitopes. Thus, linked to emerging rationally designed antigens, immunization strategies that also selectively direct the Fc domain of the induced humoral immune response may stimulate the needed maturation to induce protective immunity.

Complement has a vital role in IC delivery to FDCs (*17*, *19*, *20*); however, the specific Ab modifications that enable more effective IC capture within GCs has been less clear. A number of different cells contribute to the delivery of ICs to the GC including naïve noncognate B cells and macrophages, which typically acquire ICs via different combinations of FcRs and CRs (*35*, *61*, *62*). Previous associations between sialylated HA-specific Abs and affinity maturation (*32*) suggested that enhanced IC deposition was linked to IC signaling via CD23 (FcεRII), which is highly expressed on B cells. Although CD23 may have a role in affinity maturation (*63*), the data presented here build on our previous understanding of the critical role of complement in IC capture and deposition on FDCs and provide evidence for a mechanistic involvement of sialylation in the acceleration of antigen deposition and B cell maturation. Whether the deposited ICs accelerate affinity maturation by providing more antigen to drive higher frequencies of B cells that must compete more aggressively for limited T cell help or lead to tighter binding ICs that require B cell receptors to capture antigen more “avidly” for survival signals is unclear. It remains unknown whether the antigen specificity of the ICs influences this effect. Data from our longitudinal samples indicate that Fc interactions are higher in patients that develop neutralizing Abs even before neutralizing Abs have developed, suggesting that non-neutralizing Abs could contribute to this effect. Future studies focusing on human Abs in human Fc chimeric mice, in the presence of other mAb specificities, including those that are non-neutralizing, linked to sophisticated affinity maturation measures, may elucidate the mechanisms underlying the adjuvanting effects of the IC-Fc. Nonetheless, the data here highlight the “adjuvanting” activity of sialylated Abs in B cell maturation, pointing to potential opportunities to leverage the Fc domain to drive enhanced affinity maturation in the context of HIV vaccination.

Complement-activating Abs have also been implicated in the destruction of HIV virions (*64*). Thus, sialylation may provide a bifunctional advantage in driving enhanced affinity maturation and direct antiviral control of HIV. Although HIV can mutate rapidly to escape the selection pressure from neutralizing Abs in the context of natural infection, it is plausible that vaccines inducing complement-depositing Abs against HIV may enhance both Fab- and Fc-mediated control of the virus. Thus, with our emerging ability to directly program Ab glycosylation, via distinct immunogens (*51*) or adjuvants (*53*), next-generation vaccine strategies may harness the full antiviral breadth of the humoral immune response. Thus, coupled to sophisticated emerging immunogens, manipulation of the Fc domain of the Ab may support the evolution of neutralizing Abs that block most strains of HIV infection.

## MATERIALS AND METHODS

### Study design

The goals of this study were to identify unique properties of the Abs from individuals with bNAbs to HIV and to determine whether these properties could affect the humoral immune response. We used systems serology, computational analysis, enzyme-linked immunosorbent assay (ELISA), glycan analysis, Luminex, flow cytometry, confocal microscopy, and mouse immunization to accomplish these goals. The samples were blinded throughout the initial Fc profiling and unblinded for analysis. The number of independent experiments and inclusion of individuals is described in the figure legends and in Materials and Methods.

### Study individuals

A total of 131 controllers were included for cross-sectional studies, including both elite controllers, who spontaneously control viral replication to undetectable levels (<75 copies/ml; CD4, 353 to 1813 cells/mm^3^), and viremic controllers with detectable but low viral loads (~20 to 1658 copies of RNA/ml; CD4, 172 to 1794 cells/mm^3^) (*15*). Days after diagnosis of HIV range from 1 to 30 years, with similar distributions across neutralizers and non-neutralizers. All individuals signed informed consent, and the study was approved by the Massachusetts General Hospital (MGH) Institutional Review Board. HIV-1 neutralization breadth was assessed using the TZM-bL cell–based pseudovirus neutralization assay (*65*) against a panel of Env pseudoviruses derived from nine clade B tier 2 (AC10.0.29, RHPA4259.7, THRO4156.18, REJO4541.67, WITO4160.33, TRO.11, SC422661.8, QH0692.42, and CAAN5342.A2) and two tier 3 (PVO.4 and TRJO4551.58) neutralization sensitivities. Neutralization was defined as at least 50% inhibition of infection at a 1:20 dilution. The neutralization breadth was defined as the percentage of the 11 isolates neutralized by each plasma sample; neutralizers were able to neutralize anywhere from 1 virus (9% breadth) to all 11 viruses (100% breadth), and non-neutralizers had undetectable cross-neutralization against all 11 tier 2/3 viruses. All samples that showed reactivity to the murine leukemia virus– pseudotyped virion controls were excluded.

In addition, longitudinal plasma samples were included from 26 acutely infected individuals who were recruited as part of the San Diego Acute and Early Infectious Disease Research Program (*7*). Twelve individuals developed neutralizing Ab breadth after 2 to 3 years of infection, whereas 14 individuals (matched for age, gender, and ethnicity) did not develop any appreciable tier 2 neutralizing Ab breadth. Plasma samples were analyzed at 1 month, 6 months, and 1 year after the estimated time of infection. These limited time points were included not only to ensure that sampling occurred before the evolution of neutralizing Ab breadth but also to maintain consistency in comparison for matched durations of infection. All individuals provided informed consent at their respective institutions.

### Functional assays

#### ADCP assay

The THP-1 phagocytosis assay was performed as previously described (*66*). Briefly, THP-1 cells were purchased from the American Type Culture Collection and cultured as recommended. Biotinylated rgp120-YU2 (Immune Technology) was used to saturate the binding sites on 1-µm fluorescent neutravidin beads (Invitrogen) overnight at 4°C. Excess antigen was removed by washing, and then beads were incubated with patient Ab samples for 2 hours at 37°C. After opsonization, THP-1 cells were added and incubated overnight to allow phagocytosis. Cells were then fixed with 4% paraformaldehyde (PFA), and the extent of phagocytosis was measured via flow cytometry on a BD LSR II flow cytometer equipped with high-throughput sampler. The data are reported as a phagocytic score, which takes into account the proportion of effector cells that phagocytosed and the degree of phagocytosis [integrated mean fluorescence intensity (MFI): frequency × MFI]. Each Ab sample was tested over a range of concentrations (0.1 to 100 µg/ml).

#### ADCC assay

The rapid fluorescent ADCC assay was performed as previously described (*67*). Briefly, CEM-NKr cells were pulsed with rgp120-YU2 proteins (6 µg/ml) and labeled with the intracellular dye carboxyfluorescein diacetate succinimidyl ester (CFSE) and the membrane dye PKH26. NK cells were enriched directly from seronegative donor whole blood by negative selection using RosetteSep (STEMCELL Technologies). Purified IgG was added to the labeled, antigen-pulsed CEM-NKr cells before the addition of fresh NK cells. The cells were incubated for 4 hours at 37°C and then fixed with 4% PFA. The proportion of cells that maintained membrane expression of PKH26 but lost CFSE staining (i.e., lysed cells) were quantified via flow cytometry.

#### Ab-dependent complement deposition

ADCD was assessed by the measurement of complement component C3b on the surface of target cells. CD4-expressing target cells were pulsed with the rgp120-YU2 protein and incubated with serum Abs. Freshly isolated HIV-negative donor plasma diluted into veronal buffer with 0.1% gelatin (1:10 dilution) and the cells were incubated for 20 min at 37°C. The cells then were washed with 15 mM EDTA in phosphate-buffered saline (PBS), and complement deposition was detected via flow cytometry after staining for C3b (Cedarlane). Replicates using heat-inactivated donor plasma were used as negative controls.

#### IC uptake by Raji B cells and primary human naïve B cells

Ab-dependent binding to CRs was assessed using CR-expressing Raji cells (*68*) or naïve B cells from fresh peripheral blood mononuclear cells (PBMCs). Specifically, biotinylated avitag-gp120-YU2 (ImmuneTech) was combined with allophycocyanin (APC)–labeled streptavidin (Invitrogen) for 2 hours at 37°C. Serial dilutions of sialylated PGT121 or nonsialylated PGT121 Abs were added to prepare S-ICs or NS-ICs, respectively. ICs were washed with PBS + 5% bovine serum albumin (BSA) and incubated with 5 × 10^4^ Raji B cells or 10^6^ human PBMCs per well for 30 min at 37°C with or without freshly reconstituted guinea pig complement in the presence or absence of anti-FcγRII Ab. Cells were stained with a viability dye (eBioscience), and Abs against CD3, CD14, and CD19, and then fixed with 100 µl of 4% PFA before flow cytometry analysis. The percentage of bead^+^ Raji B cells or live^+^CD3^−^CD14^−^CD19^+^ cells were enumerated across different conditions.

### Luminex subclass assay, FcγR, and complement protein binding

A custom Luminex assay was used to quantify the relative concentration of HIV-specific Ab subclass levels, as previously described (*69*). Briefly, microplex carboxylated beads (Luminex) were coupled to the indicated proteins via covalent *N*-hydroxysuccinimide (NHS)–ester linkages by combining 1-ethyl-3-(3-dimethylaminopropyl)carbodiimide and NHS (Thermo Scientific) in PBS, as recommended by the manufacturer (antigens: gp140-SF162, gp120-YU2, gp41- HXBc2, p24-HXBc2, and HA-Brisbane/60/09). The coupled beads [50 µl of a solution (100 microspheres/µl) in 0.1% BSA in PBS] were added to each well of a 96-well filter plate (Millipore). The purified IgGs [50 µl of each sample diluted to IgG (200 µg/ml)] were added to five wells of the 96-well plate and incubated at 4°C overnight. The beads were washed three times with 100 µl of PBS–Tween 20, and individual IgG isotype detection reagents (bulk IgG, IgG1, IgG2, IgG3, and IgG4) conjugated to phycoerythrin (PE) (Southern Biotech) were added individually to each of one of the five wells. The 96-well plate was incubated with shaking for 2 hours, washed three times, and read on a Bio-Plex 200 instrument. Similarly, FcγRIIA, FcγRIIB, FcγRIIIA, FcγRIIIB, and C1q were biotinylated (Pierce 21331), conjugated to PE (Southern Biotech), incubated with coupled beads and purified IgG, and analyzed on a Bio-Plex 200 instrument to test the FcR and complement protein binding capacity of HIV-specific Abs, as previously described (*70*).

### Glycan analysis

The relative abundance of Ab glycan structures was quantified by capillary electrophoresis, as previously described (*39*). Briefly, antigen-specific Abs were purified using gp120-coupled magnetic beads (New England Biolabs) and then treated with IdeZ protease (New England Biolabs) to collect the Ab Fc portion. N-glycans were removed from the Fc domains and labeled with 8-aminopyrene-1,3,6-trisulfonic acid (APTS) using the GlycanAssure APTS Kit (Thermo Fisher), as described in the manufacturer’s protocol. Labeled glycans were loaded onto the 3500 Genetic Analyzer (Thermo Fisher). Peaks of five major glycan structures (G0, G1, G2, bisecting, and sialylated) were identified. The relative abundance of each glycan structure was determined by calculating the area under the curve, normalized for equal amounts of loaded APTS dye, of each peak divided by the total area of all peaks.

### Purification of Ab glycoforms and ICs

PGT121 mAb was used to generate agalactosylated, galactosylated nonsialylated, and galactosylated sialylated Abs and then formulated with gp120 to prepare G0-ICs, NS-ICs, and S-ICs, respectively. Sialylated PGT121 mAb was used to generate Ïnonsialylated and agalactosylated Abs. The Ab was incubated overnight at 37°C with neuraminidase (Millipore Sigma) or galactosidase S (New England Biolabs) to generate nonsialylated or agalactosylated Abs, respectively. Glycoprofiles of the modified mAbs were confirmed by glycan sequencing (fig. S3A) and by Western blot using *Sambucus nigra* agglutinin (SNA)–biotin that binds to sialic acid. Fc glycoform composition without neuraminidase treatment was 13.14% sialylated (G2S1F) and 86.86% neutral (G0F, G1F, G1FB, and G2F). Sialylated or galactosylated glycoforms were not detected after neuraminidase or galactosidase S treatment, respectively. Neuraminidase-treated nonsialylated PGT121 retained its ability to bind efficiently to gp120 by ELISA (fig. S3B) and surface plasmon resonance (fig. S3C), as previously described (*71*).

### IC preparation

To separate the effects of elevated Ab titers from adjuvanting effects of unique Fc domains in the neutralizers, we selected polyclonal pools of Abs across the neutralizers and non-neutralizers that had equivalent titers and avidity. Specifically, gp120-specific binding titers and avidity were assessed across the top 10 neutralizers and the bottom 10 neutralizers (fig. S1A). Although gp120-specific titers were higher among the neutralizers, four individuals were selected from each group that had equivalent gp120-specific titers and avidity (encircled in red). Binding curves were performed to define the optimal concentration (1:30) of plasma to achieve equivalent bead occupancy (fig. S1, B and C).

For the preparation of ICs, biotinylated avitag-gp120-YU2 (ImmuneTech) was combined with APC-labeled or unlabeled streptavidin (Invitrogen) for 2 hours at 37°C. Polyclonal pools of neutralizer/non- neutralizer Abs were added at a ratio of 1:30. For mAb experiments, G0-PGT121, S-PGT121, or NS-PGT121 Abs were added at a ratio of 1:2 antigen/Ab and incubated for 2 hours at 37°C. All ICs were then incubated with 2:1 (antigen + Ab)/alum for 30 min at room temperature and finally brought to a final volume of 100 µl per mouse with PBS. ICs were tested for endotoxin before immunization using a *Limulus* Amebocyte Lysate test (Thermo Fisher) according to the manufacturer’s instructions and were found to have comparable low/undetectable levels of endotoxin in each sample.

### Mice and vaccinations

Pathogen-free BALB/c female mice (the Jackson Laboratory) aged 6 weeks were used in this study. All animal studies were carried out in compliance with current regulations of the MGH Center for Comparative Medicine at the Ragon Institute animal facility. To measure the frequencies of gp120^+^ immune cells by flow cytometry and to detect the B cell follicles by confocal microscopy, mice received one intravenous vaccination with fluorescently labeled ICs in PBS. To measure the gp120-specific Ab and B cell responses, mice received two intramuscular vaccinations with alum-adjuvanted ICs 3 weeks apart, and then analysis was performed 10 days after the last immunization. All formulations were adjusted with PBS to bring up to 100 µl per mouse. *C1q*^−/−^ (*72*) and WT mice in a C57BL/6 background were used to assess the role of the C1q complement protein after vaccination; these mouse experiments were approved and performed according to the guidelines of the Washington University School of Medicine Animal Safety Committee.

### Flow cytometry

For the characterization of the innate and the GC B cell responses, cell suspensions prepared from spleen, or pairs of draining inguinal lymph nodes from each mouse, were stained with blue live/dead cell stain (Invitrogen) for 20 min at room temperature and then incubated with Fc block (BD Biosciences) in PBS plus 1% fetal bovine serum (HyClone, Thermo Scientific) for 10 min at 4°C. About 10^7^ splenocytes or 2 × 10^6^ to 3 × 10^6^ lymph node cells were stained for 30 min at 4°C with the following mAbs: anti-CD19 APC-H7 (BD Biosciences), anti-CD3 BV785 (BioLegend), anti-CD38 peridininchlorophyll- protein complex (PerCP)–C5.5 (BD Biosciences), and anti-CD95 BV510 (BD Horizon). Cells were analyzed on a FACS Fortessa (BD Biosciences), and data analyses were performed using FlowJo software v9.6 (Tree Star).

### Mouse Ab titers and avidity measurement

Titration of gp120-specific serum IgG was performed on individual serum samples with ELISA plates (Nunc MaxiSorp, Thermo Scientific) coated overnight with gp120-YU2 (250 ng/ml). Plates were blocked with 2% BSA in PBS/0.05% Tween 20 for 1 hour at 37°C and then washed three times with 0.05% Tween 20 in PBS and incubated for 2 hours at 37°C with individual mouse sera in twofold serial dilutions. Plates were washed, incubated for 1 hour at 37°C with secondary anti-mouse total IgG (1:2000; Sigma-Aldrich), and washed, and 3,3ʹ,5,5ʹ- tetramethylbenzidine was added for detection. The color reaction was measured with a Tecan reader by determining the optical density at 450 nm (OD_450_), which was used to plot the graphs. Avidity was measured as the titer of high-avidity Abs that were not washed away after plate washing with 7 M urea for 15 min before the addition of the secondary Ab (*73*). The ratio of gp120-specific high-avidity Abs to gp120-specific total Abs was used to compare groups with different gp120-specific total Ab titers.

### Confocal microscopy

Spleens were collected from immunized animals at the appropriate time points and cut into ~5-mm-thick pieces, immediately submerged in optimal cutting temperature (O.C.T.) compound (Tissue-Tek, Sakura), snap-frozen using 2-methylbutane (isobutene) and liquid nitrogen, and stored in liquid nitrogen until processing. The cryosections (40 µm thick) were cut along the entire organ to analyze all planes. The cryosections were fixed using ice-cold acetone for 10 min at room temperature and rehydrated in PBS for 5 min. Sections were then blocked by Background Sniper (Biocare) and stained using anti-CD35 (IgG-purified, BD Pharmingen) or anti–GL-7 (IgM-purified, eBioscience), followed by rat anti-IgG Alexa568 (Abcam) or by rat anti-IgM Texas Red (Sigma), respectively. Sections were also stained with anti-CD169 [fluorescein isothiocyanate (FITC), BioLegend]. The antigen APC-labeled gp120 used for the immunizations was detected. After washing three times with PBS, stained tissue sections were sealed using Gold Antifade reagent (Invitrogen–Life Technologies) and a coverslip. Images were acquired with a Zeiss LSM 510 confocal microscope.

### Statistical analysis

PLS-DA defined the relationship between the input as a linear combination of all features among neutralizers or non-neutralizers (*33*). PLS-DA seeks the latent variables, which linearly combine all features, that explain the maximum variance between the groups; this approach generates a model with the greatest separation among groups with the lowest possible mean squared error. Before PLS-DA, the data were normalized with mean centering and variance scaling; 10-fold cross-validation was performed by iterative random exclusion of subsets of sample data (in groups of 10%) during model calibration, and then the excluded data were used to test model predictions. Ultimately, each variable identified in the model was weighted for its contribution to splitting the groups, aimed at generating a variable importance in projection.

Analysis of variance (ANOVA), followed by Tukey’s, Dunnett’s, or Bonferroni’s post tests, was performed using GraphPad Prism 7 software (GraphPad Software) to compare differences between more than two groups. A Spearman correlation, followed by Bonferroni correction, was used to assess the linear relation between breadth and FcR/C1q binding Abs. Mann-Whitney and Student’s *t* tests were used to compare differences between groups using GraphPad Prism 7. Spider plots were generated with Microsoft Excel 2011.

## SUPPLEMENTARY MATERIALS

immunology.sciencemag.org/cgi/content/full/3/26/eaat7796/DC1

Fig. S1. Process of selection of plasma samples and dilutions for IC immunization.

Fig. S2. Titers of serum HIV-specific and flu-specific Ab subclasses.

Fig. S3. Correlation of IgG gp120-specific titers and neutralization breadth to CD4 count, days

after diagnosis, viral load, or controller status.

Fig. S4. Binding and glycosylation profiles of modified PGT121 monoclonals.

Table S1. Cohort neutralization profiles.

Table S2. Raw data sets.

## Data and materials availability

All data needed to evaluate the conclusions in the paper are present in the paper or the Supplementary Materials.

**Citation** G. Lofano, M. J. Gorman, A. S. Yousif, W.-H. Yu, J. M. Fox, A.-S. Dugast, M. E. Ackerman, T. J. Suscovich, J. Weiner, D. Barouch, H. Streeck, S. Little, D. Smith, D. Richman, D. Lauffenburger, B. D. Walker, M. S. Diamond, G. Alter, Antigen-specific antibody Fc glycosylation enhances humoral immunity via the recruitment of complement. *Sci. Immunol.***3**, eaat7796 (2018).
