# Supplementary material for: Antigen-specific antibody Fc glycosylation enhances humoral immunity via the recruitment of complement
Source: Sci Immunol. 2018 Aug 17;3(26):eaat7796. doi: 10.1126/sciimmunol.aat7796 (PMC6298214; doi:10.1126/sciimmunol.aat7796)
Supplement: Supplementary file 1 [file SI-03-eaat7796-s1.pdf]

Science Immunology
AAAS

immunology.sciencemag.org/cgi/content/full/3/26/eaat7796/DC1

Supplementary Materials for

Antigen-specific antibody Fc glycosylation enhances humoral immunity via the recruitment of complement

Giuseppe Lofano, Matthew J. Gorman, Ashraf S. Yousif, Wen-Han Yu, Julie M. Fox, Anne-Sophie Dugast, Margaret E. Ackerman, Todd J. Suscovich, Joshua Weiner, Dan Barouch, Hendrik Streeck, Susan Little, Davey Smith, Douglas Richman, Douglas Lauffenburger, Bruce D. Walker, Michael S. Diamond, Galit Alter*

*Corresponding author. Email: galter@partners.org

Published 17 August 2018, *Sci. Immunol.* **3**, eaat7796 (2018)
DOI: 10.1126/sciimmunol.aat7796

The PDF file includes:

Fig. S1. Process of selection of plasma samples and dilutions for IC immunization.
Fig. S2. Titers of serum HIV-specific and flu-specific Ab subclasses.
Fig. S3. Correlation of IgG gp120-specific titers and neutralization breadth to CD4 count, days after diagnosis, viral load, or controller status.
Fig. S4. Binding and glycosylation profiles of modified PGT121 monoclonals.
Table S1. Cohort neutralization profiles.

Other Supplementary Material for this manuscript includes the following:

(available at immunology.sciencemag.org/cgi/content/full/3/26/eaat7796/DC1)

Table S2 (Microsoft Excel format). Raw data sets.

Supplementary Figure 1

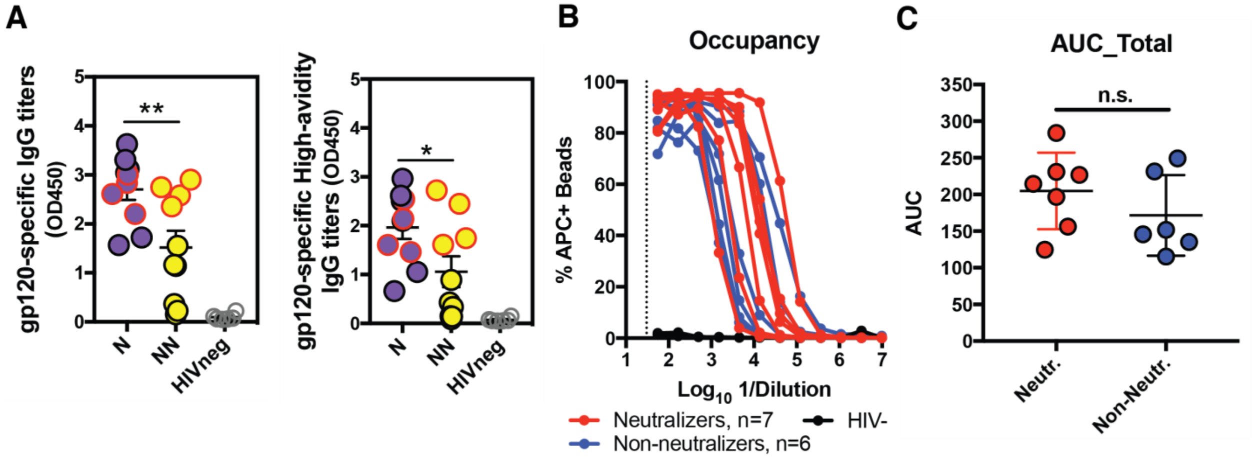

Fig. S1. Process of selection of plasma samples and dilutions for IC immunization. (**A**) The dot plot depicts the gp-120 specific antibody titers of the subjects for who sufficient plasma was available to form immune complexes. Subjects circled in red were selected for the immune complex studies due to similar titers, avidity, and availability of serum. N=10-6. (**B**) The line graph shows the overall occupancy profiles of all the neutralizers and non-neutralizers for who sufficient serum was available. At a 1:30 dilution (dotted line), no significant differences in occupancy should be detected as the majority of the beads have reached maximum occupancy. Each line is a separate serum sample, n=7-6. (**C**) Area under the curve analysis of (B) also demonstrates that there are not significant differences in the occupancy profiles of neutralizers and non-neutralizers. n=7-6. One-way ANOVA with Turkey's multiple comparison was used in A, and an unpaired t test was used in C for statistical analysis. *p<0.05, **p<0.01. The horizontal bars in all panels indicate mean, and error bars in all panels represent SEM.

Supplemental Figure 2

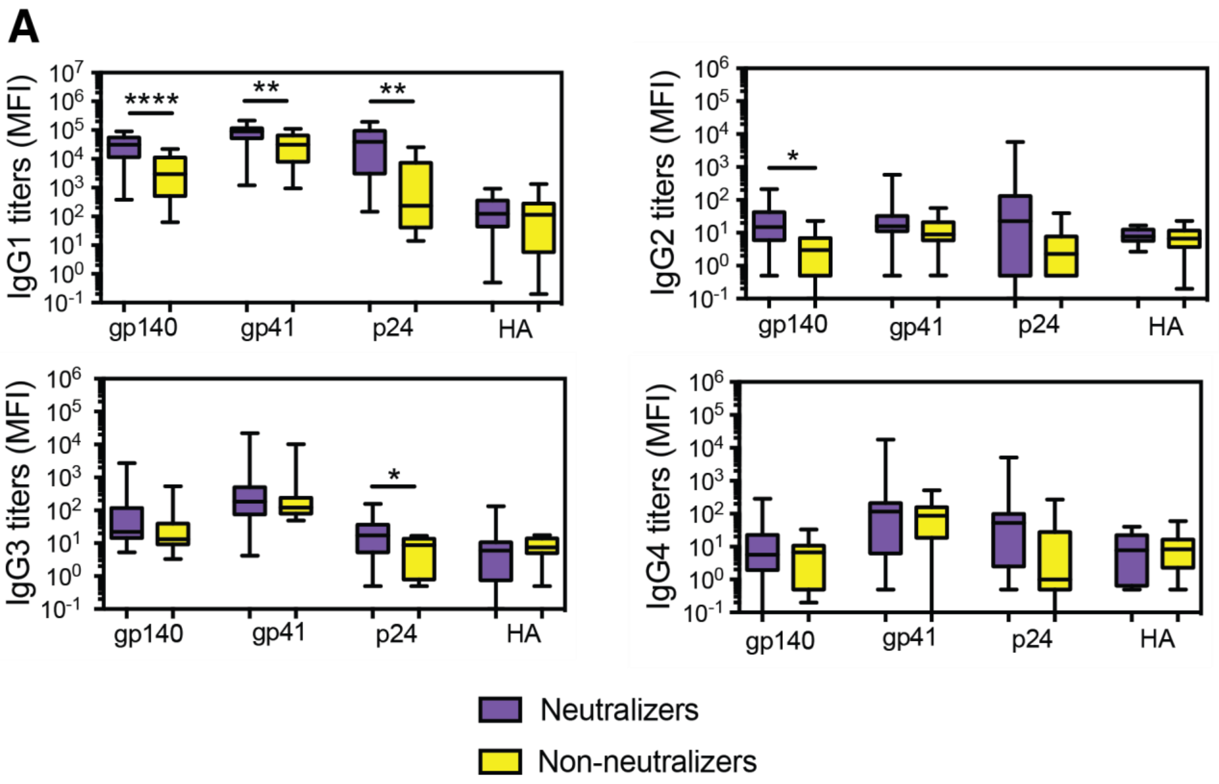

Fig. S2. Titers of serum HIV-specific and flu-specific Ab subclasses. (**A**) The titers of HIV-specific IgG1, IgG2, IgG3 and IgG4 subclasses were measured for gp140, gp41 and p24 HIV antigens by luminex assay. Flu-specific IgG1, IgG2, IgG3 and IgG4 subclass titers were also measured for the Hemagglutinin (HA) antigen. Purple boxes indicate Neutralizers, yellow boxes indicate Non-Neutralizers. n=29 neutralizers, n=15 non-neutralizers. Unpaired t tests were used for statistical analysis. *p<0.05, **p<0.01, ***p<0.001, ****p<0.0001. The whiskers represent min to max values.

Supplemental Figure 3

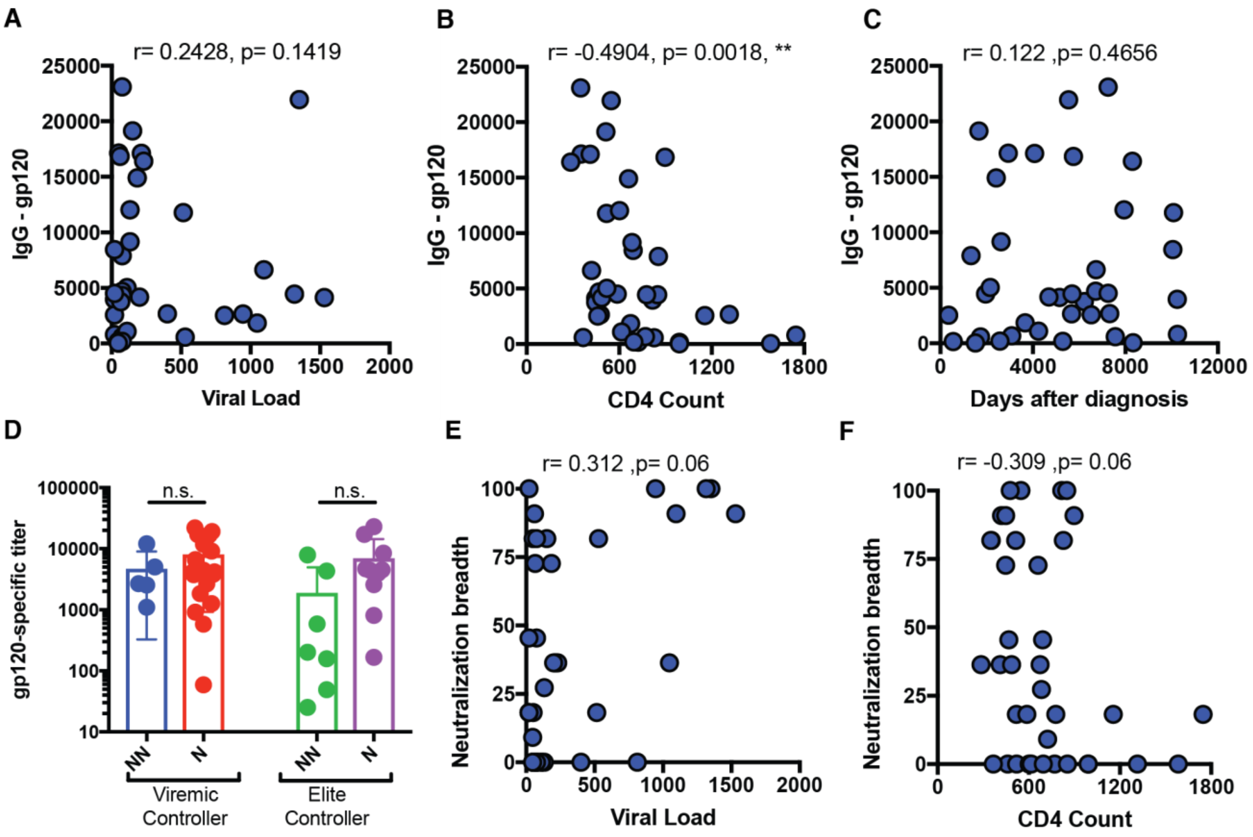

Fig. S3. Correlation of IgG gp120-specific titers and neutralization breadth to CD4 count, days after diagnosis, viral load, or controller status. (**A-C**) The dot plots show the relationship between gp120-specific titers to viral load, CD4 count, and days since diagnosis among controllers in this study. n=38. (**D**) The dot plot highlights the lack of a difference in gp120-specific antibody titers among neutralizers and non-neutralizers among the different Controllers: Viremic Controllers (VC: <50-2000 RNA copies and elite controllers: <50 copies RNA). n=5-20. (**E, F**) The dot plots show the relationship between neutralization breadth to viral load and CD4 count among controllers in this study. n=38. Spearmen correlation and an unpaired t-test was used for statistical analysis. *p<0.05, **p<0.01, **p<0.001. The horizontal bar indicates mean and the error bars represent SD in (D).

Supplementary Figure 4

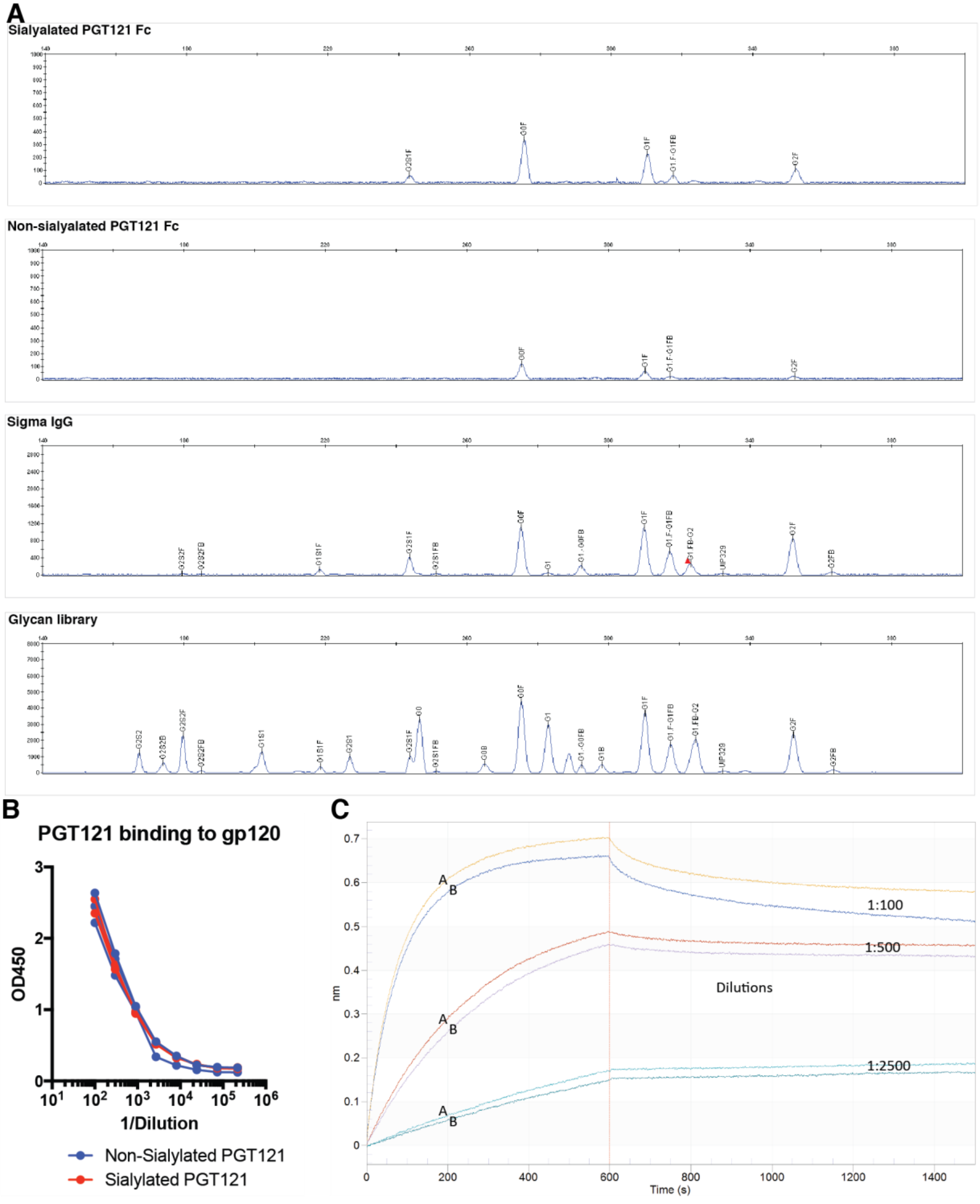

D

Sample	k_{ON} (1/Ms)	k_{OFF} (1/s)	K_D (M)	$K_{D(A)}$ / $K_{D(B)}$
A- Sialylated PGT121	2.14E+05	2.02E-04	9.43E-10	1.1
B- Non-Sialylated PGT121	2.00E+05	1.61E-04	8.06E-10	

Fig. S4. Binding and glycosylation profiles of modified PGT121 monoclonals. (**A**) Glycan analysis was performed to confirm the specific removal of sialylation. The capillary electrophoretic traces highlight the presence of sialylated peaks in the unmodified fraction (top), the loss of the sialic acid peaks in the enzymatically treated antibody (second from top), and the presence of clear sialic peaks in our 2 control standards sigma IgG (second from bottom) and the commerical standards (bottom). (**B**) ELISA binding was performed on glycan-optimized PGT121 variants to confirm the lack of a difference in sialylated PGT121 (red) and non-sialylated PGT121 (blue) binding to gp120. (**C**) SPR analysis highlights the lack of a difference in sialylated or non-sialylated PGT121 binding to gp120. (**D**) The table summarizes the results from the SPR analysis of sialylated PGT121 and non-sialylated PGT1212 binding to gp120.

Supplementary Table 1

Table S1. Cohort neutralization profiles. This table shows the sample ID number, days after diagnosis, CD4 count, viral load, and neutralization profile of the 26 neutralizing and 12 non-neutralizing serum samples used in this study against a panel of 9 tier 2 viruses (AC10.0.29, RHPA4259.7, THRO4156.18, REJO4541.67, WITO4160.33, TRO.11, SC422661.8, QH0692.42, CAAN5342.A2), 2 tier 3 viruses (PVO.4 and TRJO4551.58), and a negative (MuLV) control. These 38 serum samples were chosen from the original 131 participants due to availability of serum for further study.

Sample ID	Neutralization	Days after diagnosis	CD4 count	Viral Load	QH0692.42	SC422661.8	PVO.4	TRO.11	AC10.0.29	RHPA4259.7	THRO4156.18	REJO4541.67	TRJO4551.58	WITO4160.33	CAAN5342.A2	MuLV	Breadth %
1	N	10243	817	20	130	639	309	498	1462	287	346	851	279	134	207	28	100
2	N	5549	548	1350	170	597	217	264	167	653	203	919	206	182	273	20	100
3	N	1955	850	1315	67	162	235	420	136	1094	81	433	149	227	106	20	100
4	N	5671	478	945	479	507	919	376	395	674	308	328	160	276	771	49	100
5	N	6736	421	1095	221	285	689	230	152	196	174	1225	44	147	175	38	91
6	N	5151	447	1530	263	438	1017	332	1689	559	64	358	132	201	178	24	91
7	N	5756	898	60	435	1161	1430	778	223	2582	67	263	144	457	93	28	91
8	N	1667	513	151	530	772	507	137	70	913	143	190	120	127	44	36	82
9	N	2930	353	50	151	120	227	405	96	351	84	355	204	42	143	30	82
10	N	1735	827	529	150	188	102	471	247	90	21	326	30	345	503	26	82
11	N	7256	350	75	110	257	20	211	231	111	169	277	64	143	220	35	82
12	N	2411	661	184	339	218	294	138	31	380	78	252	27	153	42	20	73
13	N	6222	447	66	298	1370	926	582	71	2625	232	416	160	80	120	50	73
14	N	6719	468	75	20	262	295	162	71	76	224	138	28	221	225	70	45
15	N	10052	690	20	67	76	20	160	186	170	20	20	28	46	20	20	45
16	N	4075	411	214	205	20	57	84	20	49	155	165	20	36	41	26	36
17	N	3667	674	1047	231	44	51	235	32	674	96	38	28	91	196	65	36
18	N	8309	285	230	121	44	68	128	196	116	88	717	69	609	161	53	36
19	N	4691	486	200	23	190	20	91	179	72	110	251	74	78	232	38	36
20	N	2623	683	132	352	188	105	411	64	118	69	494	129	112	300	105	27
21	N	10088	518	515	44	93	20	47	55	42	20	93	41	26	20	20	18
22	N	5690	778	50	49	195	46	74	95	127	115	171	20	98	64	56	18
23	N	10258	1746	20	39	67	20	45	43	20	20	87	36	20	20	20	18
24	N	6521	1156	20	60	83	25	66	22	28	53	82	80	55	32	27	18
25	N	7254	587	20	51	191	27	146	20	20	20	20	20	47	20	20	18
26	N	5299	724	48	63	82	20	67	20	23	28	150	84	76	43	29	9
27	Non	351	461	812	63	42	20	146	59	52	20	20	217	79	124	79	0
28	Non	7327	1314	400	47	20	20	20	20	43	22	53	20	20	22	20	0
29	Non	2157	519	111	128	20	85	78	41	49	70	20	20	68	26	55	0
30	Non	3080	770	75	79	87	26	83	85	77	101	49	33	156	98	150	0
31	Non	7946	602	132	52	69	90	80	48	113	118	42	65	40	46	43	0
32	Non	7560	367	75	78	166	101	86	147	97	150	131	28	115	119	105	0
33	Non	1322	854	75	64	20	50	45	36	28	42	25	20	46	47	63	0
34	Non	4242	615	110	73	20	53	58	45	55	44	97	20	21	76	35	0
35	Non	552	990	48	25	65	30	36	115	37	61	44	20	59	52	44	0
36	Non	1514	990	50	20	40	23	51	30	31	44	79	32	71	81	69	0
37	Non	2578	695	75	57	42	63	66	78	28	66	58	20	139	67	110	0
38	Non	8336	1583	48	40	59	109	67	22	33	30	24	43	51	72	45	0
